# Clinical G. I. Surgery

**Published:** 2008

**Authors:** Avinash Supe

**Affiliations:** Professor and Head, Department of Surgical Gastroenterology

Over the last two decades, there have been tremendous advances in imaging, endoscopy, and laparoscopy in the field of GI surgery. GI surgery is the newest super-specialty branch of general surgery, where expertise and high-volume centers have made a difference in outcomes of surgery. This has made complex surgeries possible with low morbidity and mortality rates and hence recognized by patient communities. With the rapid development of this specialty in our country, surgeons in India also now perform complex pancreatic surgery as well as liver transplants, with results as good as anywhere else. 

There are now more than 30 centers in India which provide GI surgery training. Besides, many practicing surgeons have special interest and training in the field of GI surgery. In spite of such rapid growth in this field, there is paucity of text books in this field especially in the Indian subcontinent. There are quite a few books in the international market, many of them focused on topics such as laparoscopic surgery, hepatobiliary surgery, or colorectal surgery and have emphasis on the operative aspects. Most of these books do not consider Indian situations or the socio-economic aspects. All of us were needing a comprehensive book on GI surgery in India. The text book of GI Surgery, edited by the young surgeon Dr. Haribhakti, fills this gap more than adequately. 

This comprehensive Indian book on GI surgery covers all the important subjects in a clinically relevant manner. This book gives a current and concise summary of all key topics within the specialty and concentrates on recent developments. This book is logically organized, well written, and has readable chapters on evidence-based surgical practice, histopathology, pre- and post-operative care, and a clinical approach to acute abdominal pain which comprises the introductory portion of the book. The 90 chapters of this hardcover book are organized in nine sections. Volume-1 contains sections on basic GI surgery, and Upper and Lower GI tract surgery. Volume-2 has sections on special topics such as hepatobiliary pancreatic surgery, GI bleeding, portal hypertension, and laparoscopic surgery. It also covers abdominal trauma, retroperitoneal tumors, and newer technologies. In addition to all standard topics in GI surgery, such as benign and malignant GI disorders, it also covers topics such as NCPF, EHPVO, hydatid cysts, and liver abscesses that are pertinent to our practice. Special topics such as bile duct injury and its prevention, as well as gall bladder cancer, have been written by experts in the field. I especially liked the idea of patient-oriented topics under the title of ″approach to″ jaundice, non-ulcer dyspepsia, and HIV infection. These chapters, though of varying merits, provide a broad overview. The book provides clear illustrations, discussions of accompanying anatomical and physiological changes, advice on patient management, and medical pearls. The book has given good justice to recent modalities, such as cryotherapy, ethanol ablation, radiofrequency ablation, as well as liver transplantation. The bibliographies are not encyclopedic, but include a separate, important category of suggested reading. The one deficit is the avoidable redundancy between much of the material.

There is a special chapter on practice management which covers areas such as set up, data recording, medico-legal aspects, and insurance. Though not directly relevant to the science of GI surgery, these topics would help a young surgeon to establish a good GI surgery center.

Dr. Sanjiv Haribhakti is young, well trained, and an established GI surgeon at Ahmedabad and has been able to get together eminent and prominent contributors from teaching institutions as well as corporate hospitals from all corners of the country. The editor needs to be congratulated for this stupendous task of marshalling and editing contributions from 135 authors from all over the country. This text book on Gastrointestinal Surgery brings together the national group of contributors and all experts in their respective fields, to provide a text for specialists and trainees.

Overall, as stated by the author himself, this is not a complete text book, but is intended as a reference guide for the dedicated GI surgery trainee as well as the practitioner. It is written at a senior level of comprehension and is illustrated with photographs (color as well as black and white) and line drawings. The strength of this book is the chapters written in a clinically relevant manner. If there is a drawback to the textbook, it is that there is a considerable difference in the quality of various chapters. However, because of its authoritative and diverse content, the book would be of considerable interest and benefit to all GI trainees and practitioners.

